# Supplementary figures and images for: Iroki: automatic customization and visualization of phylogenetic trees
Source: PeerJ. 2020 Feb 26;8:e8584. doi: 10.7717/peerj.8584 (PMC7049256; doi:10.7717/peerj.8584)

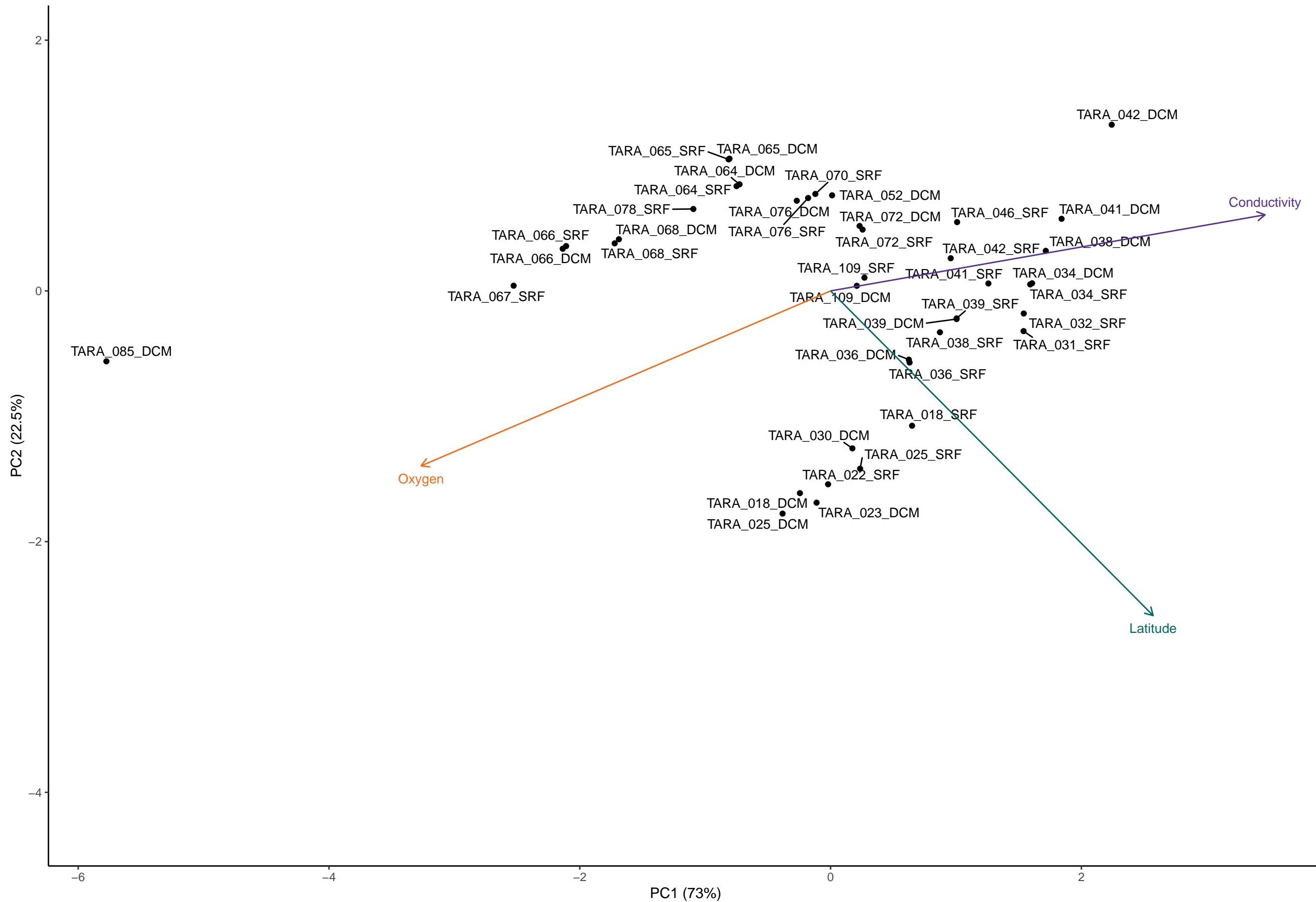

Supplement: Figure S1 — Principal components analysis biplot of 41 Tara Oceans viromes based on sample oxygen, conductivity, and latitude. [file peerj-08-8584-s002.pdf]

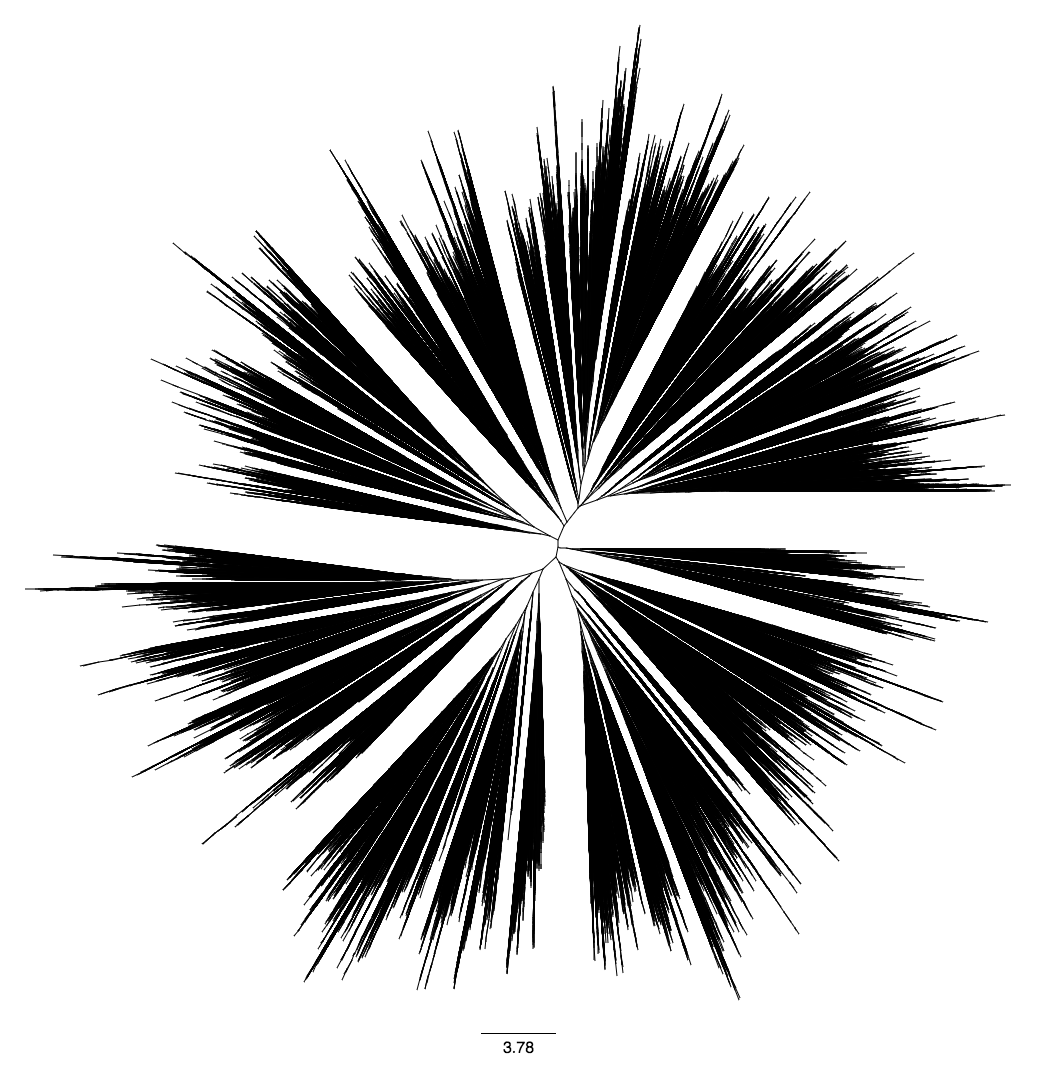

Supplement: Figure S2 — A 1,000,000 leaf tree with random branch lengths generated with rtree (using runif with default arguments for branch lengths) from the ape R package. Tree was rendered with Iroki’s Canvas tree viewer. [file peerj-08-8584-s003.png]

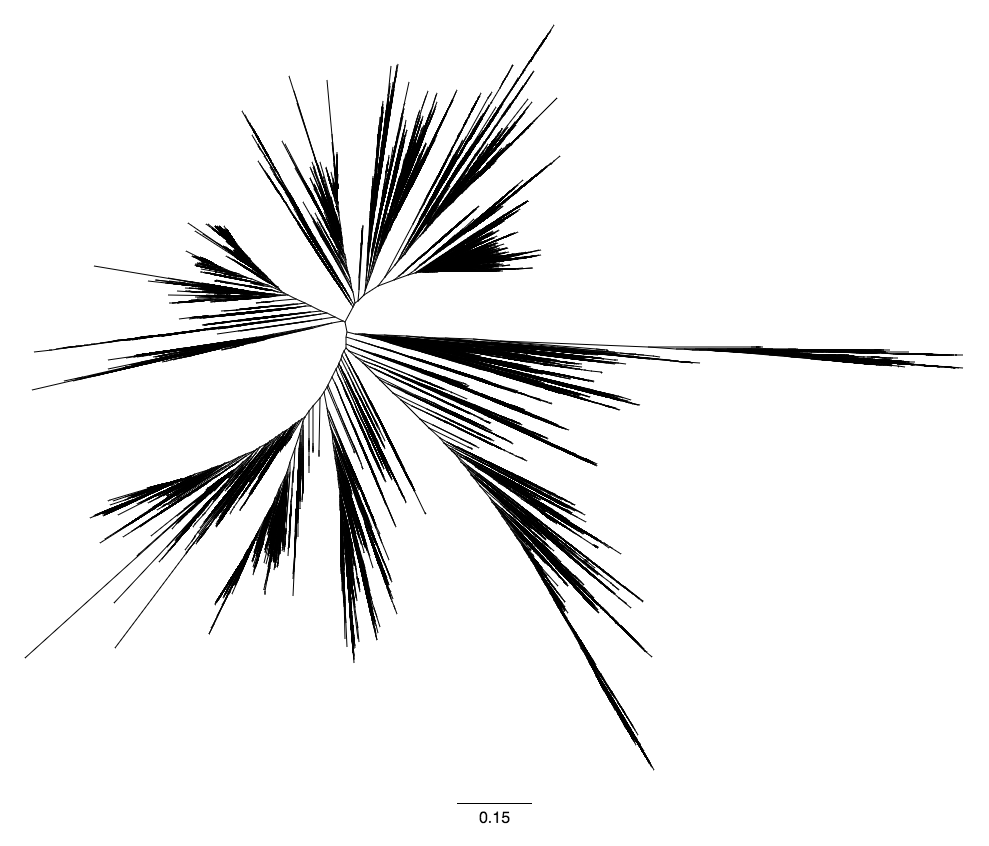

Supplement: Figure S3 — A collection of 331,550 full length SSU rRNA sequences from GreenGenes rendered with Iroki’s Canvas tree viewer. [file peerj-08-8584-s004.png]
